# Improved visualization of breast tissue on a dedicated breast PET system through ergonomic redesign of the imaging table

**DOI:** 10.1186/s13550-017-0351-7

**Published:** 2017-12-19

**Authors:** Michael K. O’Connor, Thuy D. Tran, Tiffinee N. Swanson, Lacey R. Ellingson, Katie N. Hunt, Dana H. Whaley

**Affiliations:** Department of Radiology, Charlton 1-225, Mayo Clinic, 200 First Street SW, Rochester, MN 55905 USA

**Keywords:** Dedicated breast PET, Imaging table, Breast cancer, F-18 FDG

## Abstract

**Background:**

Breast lesions closer than 2 cm to the chest wall are difficult to position in the field of view of dedicated breast PET (db-PET) systems. This inability to detect such lesions is a significant limitation of these systems. The primary objective of this study was to determine if modifications to the design of the imaging table and detector used for a db-PET system would enable improved visualization of breast tissue close to the chest wall. All studies were performed on a commercially available db-PET system (Mammi-PET). A central square section of the imaging table, containing the standard 180-mm circular aperture, was modified such that it could be removed and replaced by thinner sections with a larger aperture. Additional changes were made to the cover plate of the detector array and the patient mattress. A total of 60 patients were studied. After administration of F-18 FDG, 30 patients were imaged with a 220-mm-diameter aperture and the standard aperture, and 30 patients with a 200-mm aperture and the standard aperture. On all scans, the length of breast tissue in the field of view was measured as the greatest extent of tissue from the nipple back to the posterior edge of the breast. Image quality and patient comfort were recorded.

**Results:**

Averaged over both breasts, relative to the standard aperture, the increase in breast length was 12.5 + 7.7 mm with the 220-mm aperture, and 12.3 + 6.5 mm with the 200-mm aperture (*p* < 0.05 for both apertures). In ~ 5% of cases, the larger apertures resulted in some degradation in image quality due to closer proximity to cardiac/hepatic activity. In 10–20% of cases, movement of the breast tissue was observed as the detector ring was moved to scan the anterior region of the breast. The patient survey indicated no significant difference in the comfort level between the standard aperture and either of the prototype apertures.

**Conclusions:**

Modifications to the image table and system resulted in a significant gain in the volume of breast tissue that could be imaged on the db-PET system and should allow better visualization of lesions close to the chest wall.

## Background

Molecular imaging, particularly PET/CT, has become an important diagnostic tool over the last 10 years. Unfortunately, the development of molecular imaging systems for breast cancer has lagged behind that for other oncologic processes. One of the primary reasons for this is that many whole-body PET scanners do not appear to have adequate resolution for the detection of small (sub 10 mm) lesions in the breast [1], although this may no longer be the case with some of the latest generation of PET/CT scanners that achieve spatial resolutions of 5 mm or less [2]. Except for the staging of metastatic disease beyond the breast, PET imaging is not routinely utilized in the evaluation of primary breast cancer [3, 4].

High-resolution dedicated breast PET scanners (db-PET) have been developed over the last 10–15 years that overcome the limitations of conventional PET scanners [5–7]. These systems can achieve 1.5–2.0 mm spatial resolution in the breast, thereby allowing for reliable detection of lesions in the 3–10 mm range [8]. Earlier systems employed light compression and imaged the breast in a comparable way to mammography [5], facilitating comparison with the patient’s mammogram or tomosynthesis study. These systems required an injected activity of 370–555 MBq (10–15 mCi) F-18 FDG and generated tomosynthesis views of the breast, with high in-plane resolution, but poor resolution between slices [5]. In the latest generation of db-PET systems, the patient lies prone on an imaging table, with the breast pendulant through an aperture in the imaging table. The detector array is positioned below the table and performs a high-resolution scan of the pendulant breast. These systems provide high-resolution isotropic scans at a lower administered activity of 110–185 MBq (3–5 mCi) F-18 FDG. Prone imaging is the ideal arrangement for breast imaging as it allows for good separation between the breast tissue and the chest wall, and minimizes breathing artifact. For studies using F-18 FDG, it reduces the interference of activity in the heart and liver from that in the breast. In patients with breast tumors undergoing routine PET/CT, the prone position results in higher standard uptake values (SUV) than seen in the supine position [9].

One of the primary limitations of this design is the inability to image breast tissue close to the chest wall. A recent study by Teixeira et al. [10] found that while the db-PET system depicted 99% of primary breast cancer lesions within the scanning range of the device, the overall sensitivity dropped to 89% when primary breast lesions outside the scanning range were included. The study found that lesions closer than 2 cm to the chest wall were not usually seen on db-PET. This inability to detect such lesions is a significant limitation of the current design.

In an attempt to overcome the above limitation, we evaluated the impact of minor changes to the imaging table on the amount of breast tissue that could be imaged and on the quality of these images.

## Methods

### db-PET system

All studies were performed on a commercially available db-PET system (Mammi-PET, Oncovision, Valencia, Spain) [6, 8]. The Mammi-PET system is a dual ring detector system, with each detector consisting of 12 modules arranged in a ring with an aperture size of 186 mm. Each detector module consists of a single LYSO scintillating crystal (40 × 40 × 10 mm^3^) coupled to a position sensitive photomultiplier tube. Each ring has an effective axial field of view (FOV) of 47 mm, with the dual ring configuration providing an axial FOV of 94 mm. The detector rings move vertically in a step and shoot mode in increments of 69 mm. The detector assembly is encased in a protective 2-mm-thick fiberglass cover with a circular aperture of 180 mm.

**Fig. 1:**
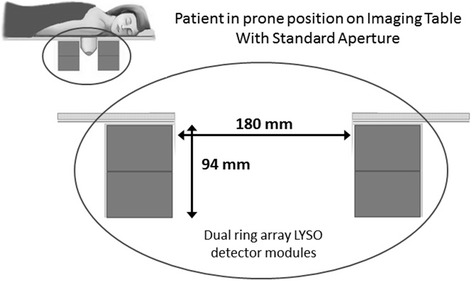
Schematic diagram showing the patient position on the db-PET imaging table, with the breast pendulant in the detector field of view. Standard aperture width = 180 mm and axial field of view = 94 mm

The associated imaging table contains a 3-mm-thick steel central section with an aperture of 180 mm diameter with the db-PET detector rings positioned directly below the aperture. In clinical use, the patient lies prone on the imaging table as illustrated in Fig. 1, with the arm of the breast being imaged, in the adducted position. The breast to be imaged is positioned over the aperture such that it is pendulant in the detector field of view. No breast compression or immobilization is used.

### Modifications to db-PET imaging table and system

**Fig. 2:**
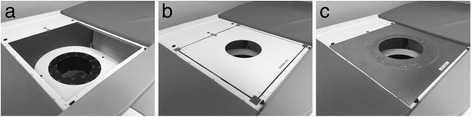
**a** Central part of the db-PET imaging table removed showing the detector ring below. **b** Central section of the table with the standard aperture repositioned as per its original configuration. **c** Aluminum plate containing the large 220-mm aperture in position over the central section of the table

For this study, a central square section of the imaging table, containing the standard aperture, was modified such that it could be removed and replaced by a section with a different aperture design. Figure 2 shows the imaging table with the central section removed (Fig. 2a), with the section containing the standard aperture (Fig. 2b), and with the large 220-mm aperture (Fig. 2c).

**Fig. 3:**
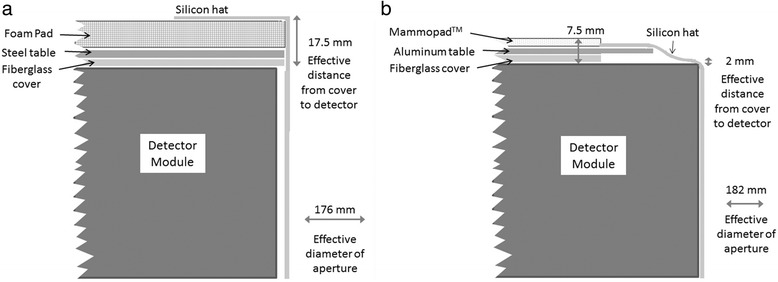
Cross-sectional schematic through part of the imaging table and detector array for **a** the standard and **b** the large prototype aperture designs. With the large aperture, the overhang from the detector cover was eliminated, table thickness was reduced, and a lighter foam pad was used

Figure 3a shows a cross-section through the standard aperture. The patient lies on a 10-mm-thick form pad (polyurethane foam material UL 94 V0) with a 180-mm aperture matched to that in the 3-mm steel table. A 2-mm-thick silicon “hat” is used to prevent contact of the breast with the walls of the detector module. A centimeter scale imprinted on the side of the silicon hat allows the technologist to estimate breast length and set the appropriate scan range for the detector ring. In addition, there is a 2.5-mm-thick fiberglass cover on the detector ring assembly with an aperture size of 180 mm. In the uncompressed mode, the overall thickness of material between the top of the table and the active detector is 17.5 mm. With compression of the foam pad, this drops to ~ 10.5 mm. The effective aperture diameter is 176 mm.

Two new aperture designs were evaluated. Both utilized 2-mm-thick aluminum sheets with aperture sizes of 200 and 220 mm, in place of the steel sheet. Aperture sizes > 220 mm were not evaluated as they would have potentially compromised table integrity. The original 10-mm foam pad was replaced with a 3-mm Mammopad (Hologic Inc., Danbury, CT) with a 270-mm aperture. This is a thinner and more compressible foam than the original pad. This pad covered the aluminum sheet up to the edge of the silicon hat. The fiberglass cover of the detector ring assembly was modified by increasing the diameter of the fiberglass aperture from 180 to 270 mm to allow greater access to the full detector field of view. Figure 3b shows a cross-section though the modified table with the large aperture. In the uncompressed mode, the overall thickness of material between the top of the table and the active detector was 7.5 mm at 5 cm from the edge of the aperture and ~ 2 mm at the edge of the aperture. The effective aperture diameter was 182 mm. The thinner plate coupled with the wider aperture and flexible silicon sleeve allowed the breast tissue to effectively sink into the detector field of view under the patient’s weight.

### Patient studies

This study was performed under an IRB-approved, HIPAA-compliant research protocol, and written informed consent was obtained from participants. A total of 60 patients, scheduled to undergo a PET/CT scan for staging or evaluation of their cancer, were recruited and assigned to group A or B (30 patients/group). Table 1 presents a breakdown of the type of cancer at presentation. Patients in group A were imaged with the 220-mm-diameter aperture and the standard aperture. Patients in group B were imaged with the 200-mm aperture and the standard aperture.

**Table 1:** Patient diagnosis at the time of the PET/CT scan

		Group A	Group B
Diagnosis	Breast cancer	5	4
	Lymphoma	10	12
	Melanoma	7	6
	Lung cancer	3	3
	Miscellaneous cancer	5	5

In our institution, patients typically receive an administered activity of 555 MBq (15 mCi) F-18 FDG with imaging commencing approximately 60–70 min post-injection. Due to its unique geometry, the db-PET system is considerably more sensitive than a conventional PET/CT system. In order to allow for decay of the F-18 to 150–185 MBq (4–5 mCi), patients were asked to return for the db-PET study approximately 3 to 3.5 h after injection of the F-18 FDG.

Each patient underwent one acquisition of each breast with the standard 180-mm circular aperture, with a scan time of 5 min per detector ring position, for a total scan time of 5–10 min/breast (depending on breast length). The central portion of the imaging table was then exchanged for one of the prototype apertures, and the acquisition repeated. The prototype table apertures were evaluated in two groups of patients. The images were reconstructed using a maximum-likelihood expectation-maximization algorithm with 16 iterations and a voxel size of 1 mm^3^. All images were corrected for random events, scatter, and attenuation through an image segmentation algorithm developed for the Mammi-PET system [11].

With both groups, immediately post-procedure, patients were asked to complete a short questionnaire to subjectively rate the relative comfort of the db-PET with the different apertures.

Responses to the following three questions were recorded using a 5-point Likert scale

1. I was comfortable during the db-PET exam with the standard table
2. I was comfortable during the db-PET exam with the prototype table
3. The amount of time it took to image each breast was reasonable

with response options of 1 = strongly disagree, 2 = disagree, 3 = neither agree nor disagree, 4 = agree, or 5 = strongly agree.

#### Image analysis

**Fig. 4:**
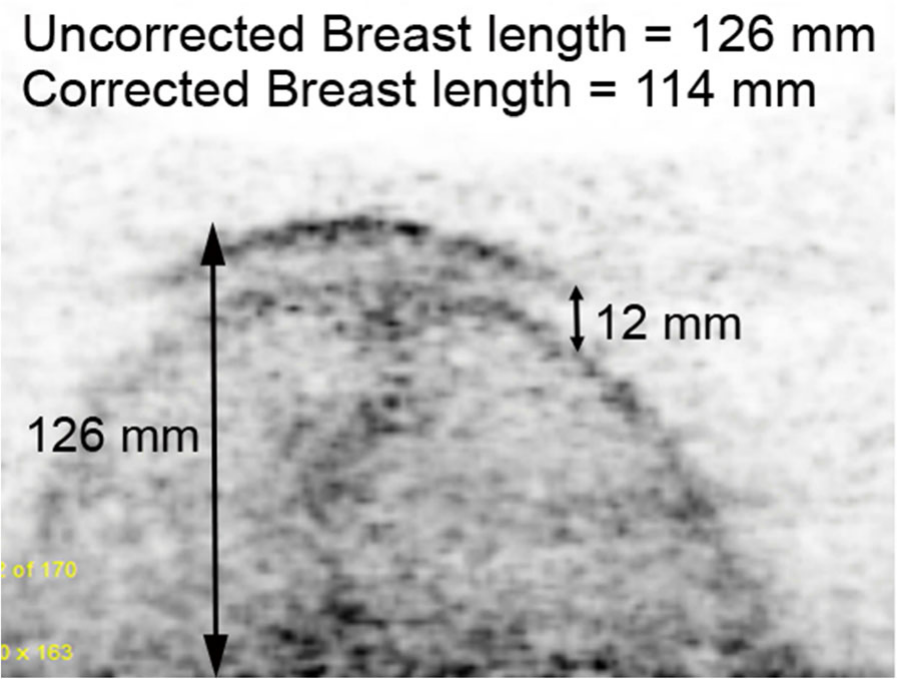
Transaxial image demonstrating a 12-mm shift of the breast as the detector rings was moved down. When present, this shift was subtracted from the measured breast length to yield the true value for breast length

In each patient, the db-PET transaxial image sets for each breast and each aperture were evaluated as follows. The length of breast tissue in the field of view was measured as the greatest extent of tissue from the nipple back to the posterior edge of the breast. With the prototype apertures, it was noted that in some patients, the larger opening allowed the breast and adjacent tissue to drop through the aperture and rest on top of the upper edge of the detector. Consequently, when the detector was moved down to image the more anterior portions of the breast, there was a corresponding drop in the breast tissue. This resulted in a double image of part of the breast, and an overestimation of true breast length. This doubling was quantified and a correction applied to the breast length. Figure 4 shows one of the more extreme examples from a study acquired with the 220-mm aperture.

**Fig. 5:**
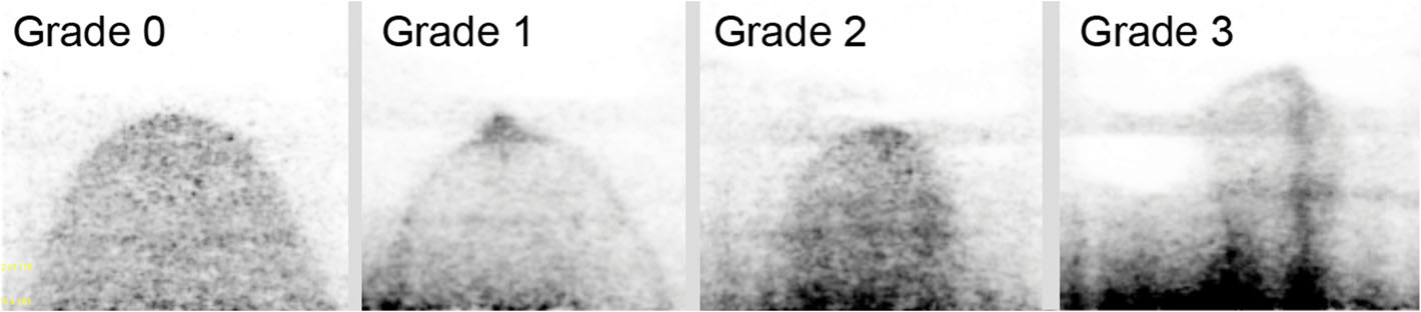
Transaxial images from 4 patient studies demonstrating increasing cross-talk from F-18 FDG in the heart into the breast images, ranging from grade 0 (no cross-talk) to grade 3 (severe increase in background activity impacting overall interpretation of the breast images)

Improved visualization of breast tissue close to the chest wall also increased proximity to the heart and liver and increased the potential for artifacts in the breast images due to the adjacency of the intense cardiac and hepatic uptake of F-18 FDG. The degree of artifact in the breast images was assessed on a 4-point subjective scale as follows: 0 = no observed impact on image quality, 1 = mild increase in background near chest wall, 2 = moderate increase in background impacting image quality near the chest wall, and 3 = severe increase in background activity impacting overall interpretation of the breast images. Figure 5 shows examples of transaxial images demonstrating each of the four categories of background interference in the breast images.

Breast length, measured using the standard and prototype apertures, was compared using a paired sample *t* test. The relative incidence of cardiac cross-talk between groups A and B was compared using a *Z*-test for two proportions. The magnitude of breast shift between the 220-mm aperture and the 200-mm aperture was compared using a *t* test calculator for two independent means. Likert scores for patient comfort, assessed using the standard and prototype apertures, were compared using a paired samples *t* test.

## Results

**Fig. 6:**
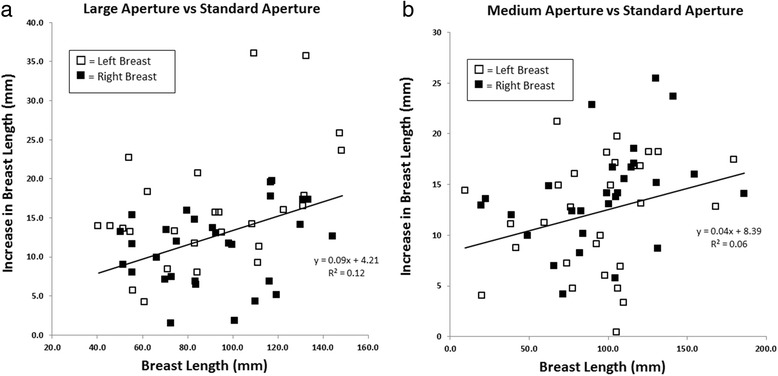
Increase in the length of breast tissue seen in the detector field of view for the **a** large—220-mm, and **b** medium—200-mm apertures, as a function of breast length measured with the standard aperture

Patient demographics and injected activity were similar for the 60 patients imaged with the two prototype apertures. Average age was 53.1 ± 13.1 years for group A and 53.1 ± 14.4 years for group B. F-18 FDG activity at the time of the scan was 171 ± 30 MBq for group A and 175 ± 32 MBq for group B. Figure 6 shows the increase in the length of breast tissue seen in the detector field of view for the 220- and 200-mm apertures, compared to the standard aperture.

Table 2 shows the measured values of breast length in the 60 patients. Averaged over both breasts, the increase in breast length was 12.5 ± 7.7 mm with the 220-mm aperture, and 12.3 ± 6.5 mm with the 200-mm aperture. For both breasts and both prototype apertures, the increase in breast length compared to the standard aperture was significant (*p* < 0.001). Scan time depends upon breast length and was 5 min for breast lengths < 90 mm, and 10 min for breast length between 90 and 160 mm. Approximately, 50% of all breasts required a 10-min scan. Only three breasts required a 15-min scan. With the 220-mm aperture, eight breasts (13%) required a longer scan time than the corresponding scan time with the standard aperture. With the 200-mm aperture, seven breasts (12%) required a longer scan time.

**Table 2:** Breast length measured from db-PET transaxial images

		Prototype aperture (mm)	Standard aperture (mm)	Difference (mm)
Group A	Left breast	103 ± 29	91 ± 27	11 ± 5
	Right breast	103 ± 36	90 ± 31	14 ± 10
Group B	Left breast	109 ± 40	96 ± 37	13 ± 6
	Right breast	105 ± 39	94 ± 38	11 ± 7

**Fig. 7:**
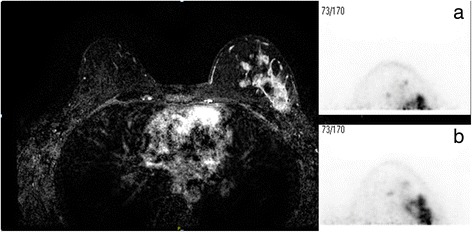
Contrast-enhanced MRI and db-PEM images of a patient with a large multi-focal breast cancer with greatest extend measured at 6.8 cm. The db-PET images acquired with the standard aperture (**a**) only show the anterior portion of the lesion, with an extent of 4.4 cm. With the large 220-mm aperture (**b**), there is more complete visualization of tumor extent (maximum extent of 6.1 cm) that closely matches that seen on the MRI

**Fig. 8:**
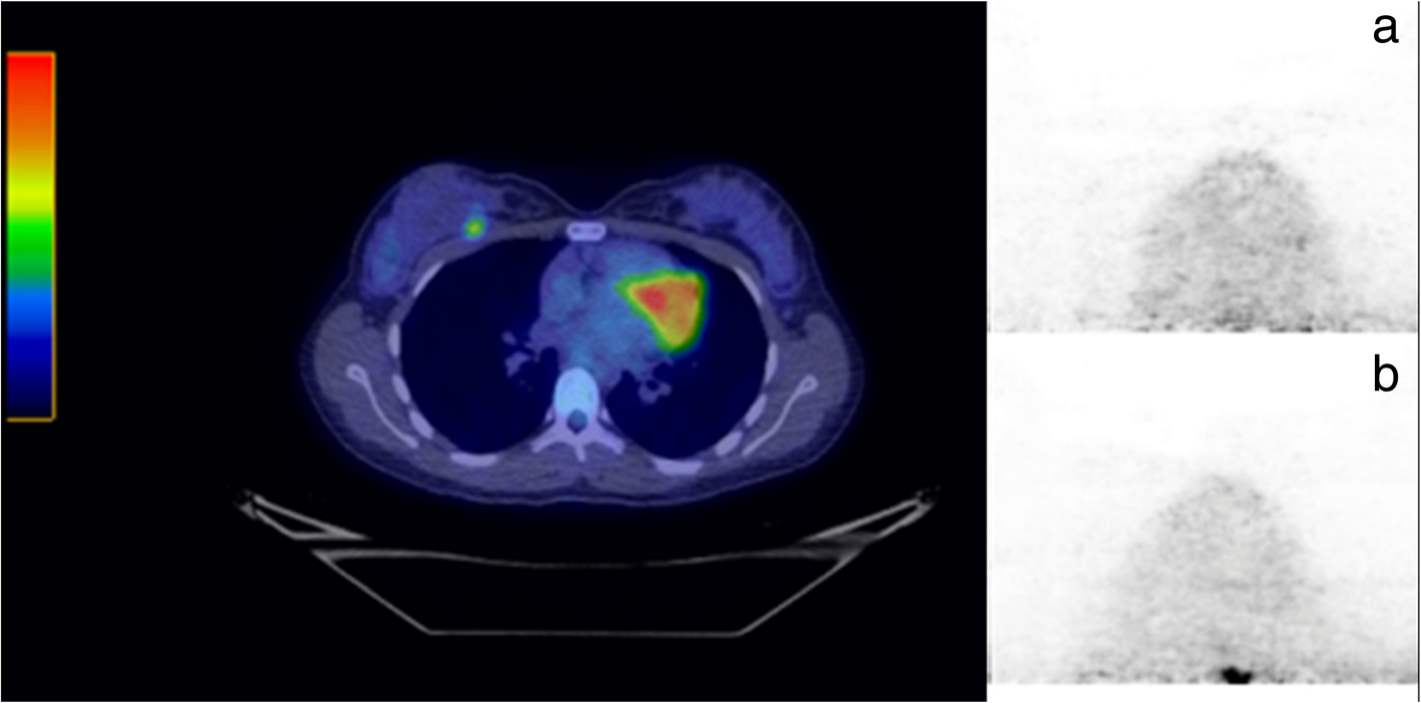
PET/CT image of a patient with metastatic lesion from malignant melanoma, located between the ribs and breast. A sub-centimeter focus of increased FDG avidity is seen within the deep soft tissues of the medial right breast. The lesion was not visible on the db-PET image acquired with the standard aperture (**a**), but can just be visualized at the posterior edge of the images acquired with the medium (200 mm) aperture, with an estimated size of 11.5 mm (**b**)

Figures 7 and 8 show examples of the increase in breast tissue obtained with the prototype apertures.

**Fig. 9:**
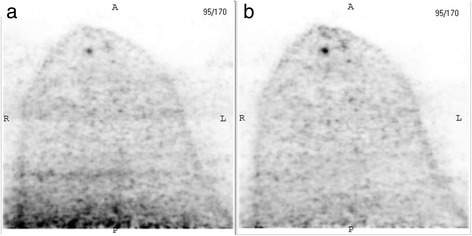
Transaxial images from a patient study demonstrating a small papilloma in the anterior portion of the breast. Cross-talk (grade 1) from non-breast F-18 FDG activity into the breast with the original reconstruction algorithm (**a**) is significantly reduced with the updated algorithm (**b**)

While the larger apertures resulted in increased visualization of breast tissue close to the chest wall, they led to an increase in two types of image artifact. These were increased interference from adjacent cardiac/hepatic activity and movement of the breast during the acquisition. The former was found to result in significant degradation in image quality in ~ 15% of cases. To minimize this effect, the manufacturer modified the reconstruction algorithm to better correct for random events close to the top of the upper detector. Figure 9 illustrates an example of a patient study before and after implementation of the new algorithm. The updated reconstruction algorithm reduced the overall incidence of studies with grade 1 and 2 cardiac cross-talk from 47 cases (38%) to 9 cases (8%) and eliminated 6 cases with grade 3 cross-talk (5%). Table 3 shows the incidence of these types of artifacts in groups A and B after implementation of the new reconstruction algorithm. There did not appear to be any correlation between the presence of cardiac cross-talk and breast movement, as no patient with grade 1 or 2 cross-talk exhibited any breast shift.

**Table 3:** Incidence of cardiac cross-talk and breast movement in db-PET images

Cardiac cross-talk	Group A		Group B	
	Std	220 mm	Std	200 mm
Grade				
0	60 (100%)	57 (95%)	60 (100%)	54 (90%)
1	0 (0%)	1 (2%)	0 (0%)	6 (10%)
2	0 (0%)	2 (3%)	0 (0%)	0 (0%)
3	0 (0%)	0 (0%)	0 (0%)	0 (0%)
Breast Shift				
0 mm	60 (100%)	47 (78%)	60 (100%)	54 (90%)
< 5 mm	0 (0%)	8 (13%)	0 (0%)	2 (3%)
5–10 mm	0 (0%)	3 (5%)	0 (0%)	2 (3%)
> 10 mm	0 (0%)	2 (3%)	0 (0%)	2 (3%)

No significant difference (*p* > 0.05) was observed in the incidence of cardiac cross-talk. While the incidence of breast shift was higher for the 220-mm aperture (overall = 21%) compared to the 200-mm aperture (9%), this difference did not reach significance (*p* = 0.14).

The patient survey indicated no significant differences in the comfort level between the standard aperture and either of the prototype apertures. With the 220-mm aperture, the Likert score on comfort was 3.6 ± 0.9 for the standard aperture and 3.5 ± 1.1 for the 220-mm aperture. Likewise, with the 200-mm aperture, the score was 3.8 ± 1.0 for the standard aperture and 3.8 ± 0.9 for the 200-mm aperture. Patients considered the length of time to acquire the images to be reasonable (Likert score = 4.2 ± 0.7 and 4.1 ± 0.7 for the 220 and 200 mm studies, respectively).

## Discussion

The higher spatial resolution of the db-PET system compared to a conventional PET/CT scanner should make the db-PET system the ideal instrument for the identification and localization of smaller breast lesions at a lower administered activity than can be achieved with PET/CT [10]. However, the advantages of a higher resolution and lower administered activity of the db-PET system are offset by the inability to detect lesions close to the chest wall. In their comparison of db-PET and PET/CT in patients with known breast lesions, Teixeira et al. [10] concluded that lesions closer than 2 cm to the chest wall were not usually detected. Average breast volume can range from 150 cm^3^ to over 2500 cm^3^, with an average value of ~ 700 cm^3^ [12]. If one assumes that the diameter of the breast close to the chest wall is comparable to the aperture opening (18 cm), then 2 cm of breast length could represent up to 500 cm^3^ of breast tissue. Therefore, small improvements in the design of the imaging table and detector may significantly increase the volume of breast tissue in the field of view and our ability to detect lesions close to the chest wall. In this study, we found that both prototype apertures combined with other changes to the padding and detector covers, resulting in ~ 12.5 mm gain in the length of breast tissue in the field of view. Assuming that the breast tissue at the chest wall completely fills the aperture, this corresponds to an additional ~ 300 mL of breast tissue (1.25 × π × 9.12) and may represent up to 40% of the volume of the breast. Whether or not this gain is adequate to allow detection of breast lesions close to the chest wall cannot be determined from this study. The examples shown in Figs. 7 and 8 clearly demonstrate the improvement obtained with the new aperture designs, but a true assessment of the improvement in sensitivity will require a repeat of the study performed by Teixeira et al. [10].

The results of the patient survey indicated that the newer aperture designs were as comfortable or uncomfortable as the standard aperture, and hence, it should be possible to implement either design into the clinical system. With all apertures, the primary complaint was the aperture digging into the rib cage. This discomfort is largely mitigated by the short acquisition time per breast (5–10 min). Our preference would be for the 200-mm aperture as it has similar gain in breast length to the 220-mm aperture and may be slightly less prone to the breast shifting between detector positions.

There were two issues that arose with the two new aperture designs. The wider opening of the aluminum plate, coupled with the silicon hat, allowed the breast to drop further into the detector ring. In some patients, this resulted in the breast tissue resting on the edges of the detector. With downward movement of the detector, this breast tissue could drop further and resulted in part of the breast being reimaged. This problem could be resolved by installing a pressure sensitive strip on the top of the detector ring. This would allow the technologist, prior to acquisition, to lower the ring until there was no contact with the breast tissue. However, this may result in the detector missing some breast tissue close to the chest wall. A better solution might be to perform post-acquisition processing to allow the technologist to re-align the transaxial slices from the first and second positions of the detector ring. This would ensure that the maximum amount of breast tissue is imaged.

The second and more significant issue was the presence of cross-talk into the breast images from F-18 FDG in the myocardium. While this was a concern with the initial reconstruction algorithm, the magnitude of the problem was greatly reduced with the updated algorithm and no case of grade 3 artifact was observed with the updated algorithm. Mild to moderate artifact (grades 1 and 2) was still observed in ~ 5% of cases. Additional improvements to the reconstruction algorithm are under development that will hopefully further reduce these artifacts.

This study has a number of limitations. We did not attempt to select patients based on breast size or body habitus; hence, it is not possible to extrapolate these findings to all patients. At this stage, we can only speculate that the use of a larger opening will improve detection of lesions close to the chest wall. Validation of this would require a repeat of the study performed by Teixeira et al. [10]. No attempt was made to determine if the changes in aperture design improved detection of lesions in the axillary tail.

Recent guidelines from the Society of Nuclear Medicine have listed the most appropriate use criteria for PET/CT in breast oncology [13]. The two clinical scenarios with the highest appropriateness score were restaging for detection of local recurrence and treatment response evaluation. With its excellent spatial resolution and improvements in its ability to image breast tissue close to the chest wall, db-PET may have a useful role in the evaluation of patients covered under these two clinical scenarios.

## Conclusions

Modifications to the image table and system resulted in a significant gain in the volume of breast tissue that could be imaged on the db-PET system and should allow better visualization of lesions close to the chest wall. Additional work is needed to eliminate minor artifacts generated by this modified design and to determine if these modifications have improved the overall sensitivity of db-PET for the detection of breast lesions.
